# Myogenic progenitor cells derived from human induced pluripotent stem cell are immune‐tolerated in humanized mice

**DOI:** 10.1002/sctm.19-0452

**Published:** 2020-09-02

**Authors:** Basma Benabdallah, Cynthia Désaulniers‐Langevin, Marie‐Lyn Goyer, Chloé Colas, Chantale Maltais, Yuanyi Li, Jean V. Guimond, Jacques P. Tremblay, Elie Haddad, Christian Beauséjour

**Affiliations:** ^1^ Centre de Recherche du CHU Ste‐Justine Montréal Québec Canada; ^2^ Centre de Recherche du CHUQ Université Laval Québec Québec Canada; ^3^ CIUSSS du Centre‐Sud‐de‐l'Ile‐de‐Montréal Montreal Québec Canada; ^4^ Département de Pédiatrie, Faculté de Médecine Université de Montréal Montréal Québec Canada; ^5^ Département de Pharmacologie et Physiologie, Faculté de Médecine Université de Montréal Montréal Québec Canada

**Keywords:** human induced pluripotent stem cells, humanized mice, myogenic progenitor cells

## Abstract

It is still unclear if immune responses will compromise the large‐scale utilization of human induced pluripotent stem cells (hiPSCs)‐derived cell therapies. To answer this question, we used humanized mouse models generated by the adoptive transfer of peripheral blood mononuclear cells or the cotransplantation of hematopoietic stem cells and human thymic tissue. Using these mice, we evaluated the engraftment in skeletal muscle of myoblasts derived either directly from a muscle biopsy or differentiated from hiPSCs or fibroblasts. Our results showed that while allogeneic grafts were mostly rejected and highly infiltrated with human T cells, engraftment of autologous cells was tolerated. We also observed that hiPSC‐derived myogenic progenitor cells (MPCs) are not targeted by autologous T cells and natural killer cells in vitro. These findings suggest that the reprogramming and differentiation procedures we used are not immunogenic and that hiPSC‐derived MPCs will be tolerated in the presence of a competent human immune system.

## Graphical abstract

Using humanized mouse models generated by the adoptive transfer of peripheral blood (Hu‐AT) or the cotransplantation of hematopoietic stem cells and human thymic tissue (Hu‐BLT), we show that the transplantation of autologous myoblast progenitor cells either derived directly from fetal muscle or differentiated from human induced pluripotent stem cells or fibroblasts were not rejected despite low level T cells infiltration.

- Myofibers derived from human induced pluripotent stem cell or fibroblasts are tolerated in autologous humanized mouse models.
- Infiltration of low‐level autologous T cells is not predictive of skeletal muscle engraftment failure.

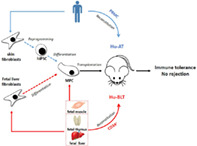

**Significance statement**

The immunogenicity of human induced pluripotent stem cell (iPSC)‐derived cells will strongly influence their use in regenerative medicine. This important feature has so far been mostly understudied given the necessity to have access to humanized mice reconstituted with an immune system autologous to iPSC‐derived cells. Using two distinct humanized mouse models, this study provides evidences that human immune cell infiltration in skeletal muscle should not be used as the sole marker to predict immunogenicity. Indeed, we show that human iPSC‐derived myogenic progenitors, similar to primary human myoblasts, are tolerated despite being infiltrated by autologous T cell. This study provides essential preclinical data supporting the usage of human iPSC‐derived myogenic progenitor cells.

## 1 INTRODUCTION

The controversial possibility that human induced pluripotent stem cell (hiPSC)‐derived cells arouse an autologous immune response could compromise their use in clinic. Indeed, Zhao and colleagues were the first to observe that mouse iPSCs, but not ESCs, are immunogenic when injected in syngeneic recipients.^1^, ^2^ Subsequently, numerous other studies have shown that mouse iPSC‐derived cells can be weakly immunogenic in syngeneic recipients and lead to the development of mechanisms similar to self‐tolerance.^3^, ^4^, ^5^, ^6^, ^7^ However, the complexity of generating humanized mouse models has limited to number of studies evaluating the immunogenicity of human iPSC‐derived cells.

Using humanized BLT mice (Hu‐BLT) generated from the cotransplantation of hematopoietic stem cells and human thymic tissue, the study by Zhao et al showed that autologous hiPSC‐derived cells can be immunogenic.^8^ They showed that hiPSC‐derived smooth muscle cells are immunogenic but that retinal pigment epithelial cells are not despite being derived from the same donor.^8^ Yet, the extent to which engrafted autologous compared to allogeneic hiPSC‐derived cells were tolerated was not evaluated in their study. Indeed, immunogenicity of muscle and retinal cells was based solely on the infiltration of autologous T cells while graft survival was not assessed. Moreover, since BLT mice are deficient in functional natural killer (NK) cells, presumably due to the absence of human IL‐15,^9^ the in vivo contribution of the innate immunity, particularly the role of NK cells in the immunogenicity of hiPSC‐derived cells was not addressed. To this end, using a combination of humanized mouse models (Hu‐BLT and mice reconstituted following the adoptive transfer [AT] of adult peripheral blood mononuclear cells [PBMCs; Hu‐AT]), we here provide evidence that skeletal muscle engraftment is not impaired despite autologous immune cell infiltration.

## 2 MATERIAL AND METHODS

### 2.1 Humanized mice

NOD/SCID/IL2Rγ null (NSG) and NSG‐SGM3 (expressing human IL3, GM‐CSF, and SCF) mice were obtained from the Jackson Laboratory (Bar Harbor, Maine) and housed in the animal care facility at the CHU Sainte‐Justine Research Center under pathogen‐free conditions in sterile ventilated racks. All in vivo manipulations were previously approved by the institutional committee for good laboratory practices for animal research (protocol #579). BLT humanized mice (Hu‐BLT) were generated by surgical implantation of small pieces (1‐2 mm^3^) of human fetal thymus tissues under the renal capsule and intravenous delivery of CD34^+^ hematopoietic stem cells isolated from autologous fetal liver into 6‐week‐old NSG mice previously irradiated with 2 Gy total body irradiation (1 Gy/min using a Faxitron CP‐160) as previously described.^10^ Fetal tissues were obtained from consented healthy donors after surgical abortion at around week 20 of pregnancy. To monitor the human immune cell engraftment in humanized mice, peripheral blood was collected and leukocytes were purified using a red blood cell lysis solution. Cells were then labeled with conjugated antibodies for human PerCP‐Cy5.5‐CD45, APC‐CD3, PE‐CD19, and FITC‐CD4 (see Table S1 for a complete list of antibodies used) and analyzed by flow cytometry (BD FACSCANTO II, BD Biosciences). For AT experiments, human adult peripheral blood was collected and immune cells purified by Ficoll (GE healthcare). Mice were injected intravenously with 1 × 10^7^ freshly isolated PBMCs.

### 2.2 Generation and characterization of hiPSCs

Fibroblasts were first isolated either from human fetal liver tissues or human skin after collagenase dissociation. Single cell fibroblasts cultures were then reprogrammed into hiPSCs using integration‐free Sendai virus (Cytotune 2.0 kit catalog # A16517 from Life Technologies). Fibroblasts were used at low population doubling (between 5 and 10) to increase efficiency of reprogramming. Emerging colonies from transduced cells were manually picked and cultured under feeder‐free conditions in Essential 8 and Essential 8 Flex medium on Geltrex‐coated dishes (Life Technologies). hiPSC clones were passaged at least 15 times to increase stable pluripotency. hiPSC generation and characterization were done in the iPSC‐cell reprogramming core facility of CHU Sainte‐Justine. hiPSC colonies were stained with the antibodies for anti‐human SSEA‐4, Sox2, OCT4, and TRA1‐60 overnight at 4°C using the pluripotent Stem Cell 4‐Marker Immunocytochemistry Kit (catalog # A24881 from Life Technologies), followed by incubation with an ALEXA secondary antibodies for 30 minutes at room temperature. Nuclei were counterstained with 4′,6‐diamidino‐2‐phenylindole (DAPI). Karyotypes were produced by G‐banding and analyzed by the CHU Ste‐Justine cytogenetic department.

### 2.3 Differentiation into myogenic progenitor cells

Myogenic progenitor cells (MPCs) were generated either directly from fibroblasts, or following fibroblast reprogramming into hiPSCs (hiPSC‐MPCs). Differentiation of fibroblasts into MPCs was obtained after transduction with a MyoD‐expressing adenovirus for 5 hours at MOI 30. hiPSC‐MPCs were differentiated by first culturing hiPSC colonies in MB1 myogenic medium (Hyclone) supplemented with 10 ng/mL of basic fibroblast growth factor (bFGF) for 5 days on Geltrex‐coated culture dishes and then transduced with a MyoD‐expressing adenovirus as described above.^11^ Both MPCs and hiPSC‐MPCs were used the day after transduction to avoid premature fusion of the cells.

### 2.4 Splenocyte and T‐cell activation assays

Splenocytes and PBMCs were isolated from Hu‐BLT mice following mechanical digestion of the spleen or isolation using Ficoll, respectively. Effector cells (splenocytes or PBMCs) were then cocultured with either autologous or allogeneic MPCs or myotubes (1 × 10^5^ or 2 × 10^5^) at 1:2 ratio during 3 days at 37°C. Effector cells were added to MPCs directly after transduction with MyoD or after a 5‐day culture in 2% fetal bovine serum to allow the formation of myotubes. T‐cell activation was measured with a phycoerythrin (PE)‐conjugated anti‐hCD69 on CD3^+^ gated viable cells by flow cytometry (BD LSRFortessa, BD Biosciences). Effector cells without stimulation were used as a negative control, and phytohemagglutinin (10 μg/mL) or an anti‐CD3 (OKT3) antibody were used as positive controls. 7‐AAD (catalog # 51.68981E from BD Biosciences) was used to exclude dead cells.

### 2.5 NK cell degranulation and cytotoxicity assays

NK cells were purified from fresh PBMCs using the NK cell enrichment negative selection kit (catalog #19055 from STEMCELL Technologies) and incubated with or without target cells at the indicated ratios. K562 cells were used as a positive control in all experiments. For the NK cell degranulation assay, effector and target cells (1 × 10^5^ or 2 × 10^5^) were cocultured at 1:2 ratio in the presence of FITC‐conjugated anti‐human CD107a/b for 1 hour at 37°C, then 2 μL/mL of monensin (catalog # 554724 from BD Biosciences) was added to the cell mixture for an additional 3 hours of incubation. For cytotoxicity assay, effector and PKH26‐stained target cells (1 × 10^4^ or 5 × 10^4^) were mixed at 1:1 or 5:1 ratio and incubated for 4 hours at 37°C. At the end of the incubation, degranulation was quantified by flow cytometry (BD LSRFortessa, BD Biosciences) after gating on CD3‐/CD56+/CD107+ viable cells and the extent of cytotoxicity was determined by the relative number of live target cells labeled with PKH26 only and dead cells labeled with both PKH26 (catalog # PKH26GL‐1KT from Sigma‐Aldrich) and 7‐AAD (BD Biosciences).

### 2.6 Fetal fibroblast and myoblast isolation

Fetal muscle and liver biopsies were minced into small pieces and digested using a solution of phosphate buffered saline (PBS) with 0.2% of collagenase (Roche) and 0.25% of dispase (STEMCELL Technologies) at 37°C during 30 minutes with manual intermittent mixing. Myoblasts and fibroblasts were then cultured and expanded respectively in myogenic MB1 medium and 10% fetal bovine serum‐supplemented Dulbecco's modified Eagle's medium (DMEM). Where indicated, only myoblasts expressing the highest level of CD56 were expanded and transplanted into mice.

### 2.7 Intramuscular transplantation of myogenic cells

*Tibialis anterior (TA)* muscle of mice were transplanted with 1 × 10^6^ myogenic cells (either myoblasts, MPCs, or hiPSC‐MPCs) in 20 μL of PBS containing 10 μg/mL of cardiotoxin (cataog# 217503‐1MG from Sigma). Of note, cardiotoxin was injected together with cells, rather than 24 hours before cell transplantation, to prevent any premature recruitment of immune cells due to muscle damage. The grafted *TA* muscles were harvested 4 weeks after transplantation and frozen in optimal cutting temperature compound (OCT, VWR). Cryosections of transplanted muscles were immunostained with anti‐human specific dystrophin (Developmental Studies Hybridoma Bank, University of Iowa) and antibodies against human CD4, CD8, and NKp46/NCR1 (all from Biolegend). For FK506 treatment, mice were injected intraperitoneally with Tacrolimus (1 mg/kg) daily. Control mice were injected with PBS.

### 2.8 MPC characterization and differentiation in vitro

MPCs cultured in MB1 were fixed and permeabilized using ethanol 95% and immunostained with a mouse anti‐human Desmin antibody from DAKO) overnight at 4°C and an anti‐mouse ALEXA fluor 594 (1:500 from Invitrogen) at room temperature for 1 hour. Confluent MPCs were maintained in DMEM supplemented with 2% fetal bovine serum and antibiotics for 3 to 5 days. Myotubes were then immunostained with a mouse anti‐Myogenin (1:500 from Abcam) or a mouse anti‐MyHC antibody (1:100 from the Developmental Studies Hybridoma Bank, University of Iowa) for 2 hours at room temperature and an anti‐mouse ALEXA fluor 594 (1:500) at room temperature for 1 hour. Nuclei were counterstained with DAPI. Cells were then phenotypically characterized by flow cytometry (BD LSRFortessa, BD Biosciences) using conjugated antibodies against HLA‐I, CD80, CD83, CD86, MICA/B, and CD112/155 (see Table S1 for a complete list of antibodies used).

### 2.9 Statistical analysis

GraphPad Prism 8 software was used for statistical analysis; *P* values on multiple comparisons were calculated using Student's *t* tests. *P < .05, **P < .01, ***P < .001, ****P < .0001.

## 3 RESULTS

### 3.1 Infiltration of autologous T cells in skeletal muscle does not mediate immune rejection in Hu‐BLT mice

**FIGURE 1:**
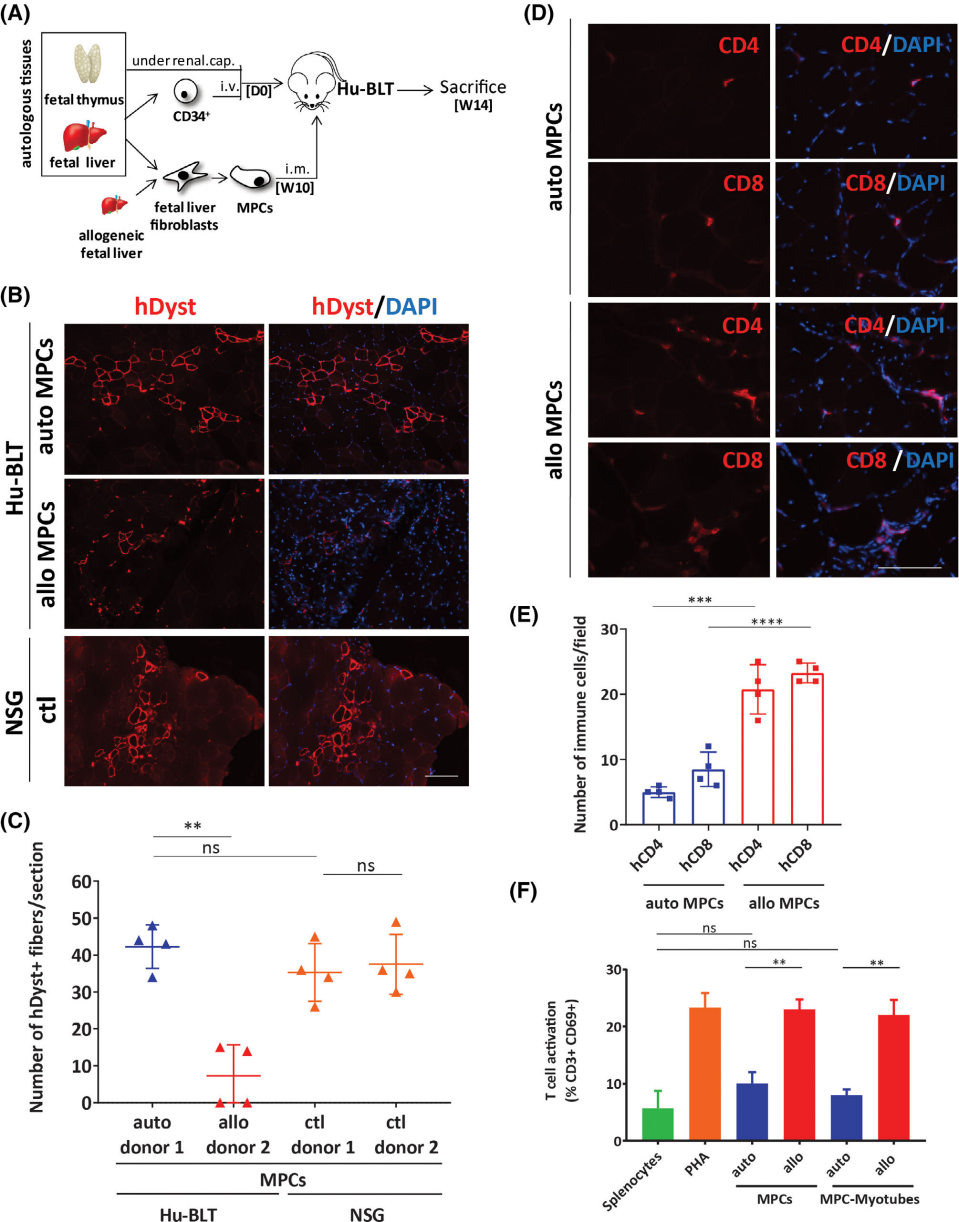
Fibroblast‐derived myogenic progenitor cells are immune tolerated in Hu‐BLT mice. A, Schematic of the BLT humanized mouse model (Hu‐BLT). NSG mice were transplanted with human fetal thymus and autologous liver‐derived CD34^+^ cells. At week 10 (W10) postimmune reconstitution, human myogenic progenitor cells (MPCs) derived from fetal liver fibroblasts were transplanted in the *Tibialis anterior* of Hu‐BLT. Mice were sacrificed 4 weeks later, and muscles were harvested and analyzed for cell engraftment and infiltration of immune cells. B, Representative photos of muscle sections from Hu‐BLT and NSG mice showing increased engraftment of autologous compared to allogeneic human MPCs and resulting human specific dystrophin‐positive fibers (in red). 4′,6‐Diamidino‐2‐phenylindole (DAPI) staining was performed to visualize nuclei (in blue). Showed are photos taken at ×20. Scale bar = 100 μm. C, Counts of human dystrophin positive fibers observed in muscle sections of Hu‐BLT mice transplanted with MPCs as shown in panel (B). Also showed are counts in nonimmune reconstituted NSG mice to assess overall engraftment potential of the different donors. Each dot represents the mean ± SEM number of dystrophin positive fibers from five selected sections in each muscle. n = 4. D, Representative photos showing CD4 and CD8 T‐cell infiltration (in red) in muscle sections of NSG (negative control) and Hu‐BLT mice transplanted with autologous or allogeneic MPCs. DAPI staining was performed to visualize nuclei (in blue). Showed are photos taken at ×40. Scale bar = 100 μm. E, Frequency of CD4 and CD8 T cells infiltrated in muscle of Hu‐BLT mice as shown in panel (D). Each dot represents the number of T cells in four randomly selected fields in each muscle. n = 4. F, Splenocytes were collected from Hu‐BLT mice 10 weeks after immune reconstitution, and the activation of T cells (as determined by gating CD3+/CD69+ cells) was measured after a coculture with autologous or allogeneic MPCs or myotubes (ratio 1:2) for 3 days. PHA was used as a positive control. Shown is the mean ± SEM of two independent experiments done in triplicate using splenocytes collected from two different mice. MPCs, myogenic progenitor cells; PHA, phytohemagglutinin

**FIGURE 2:**
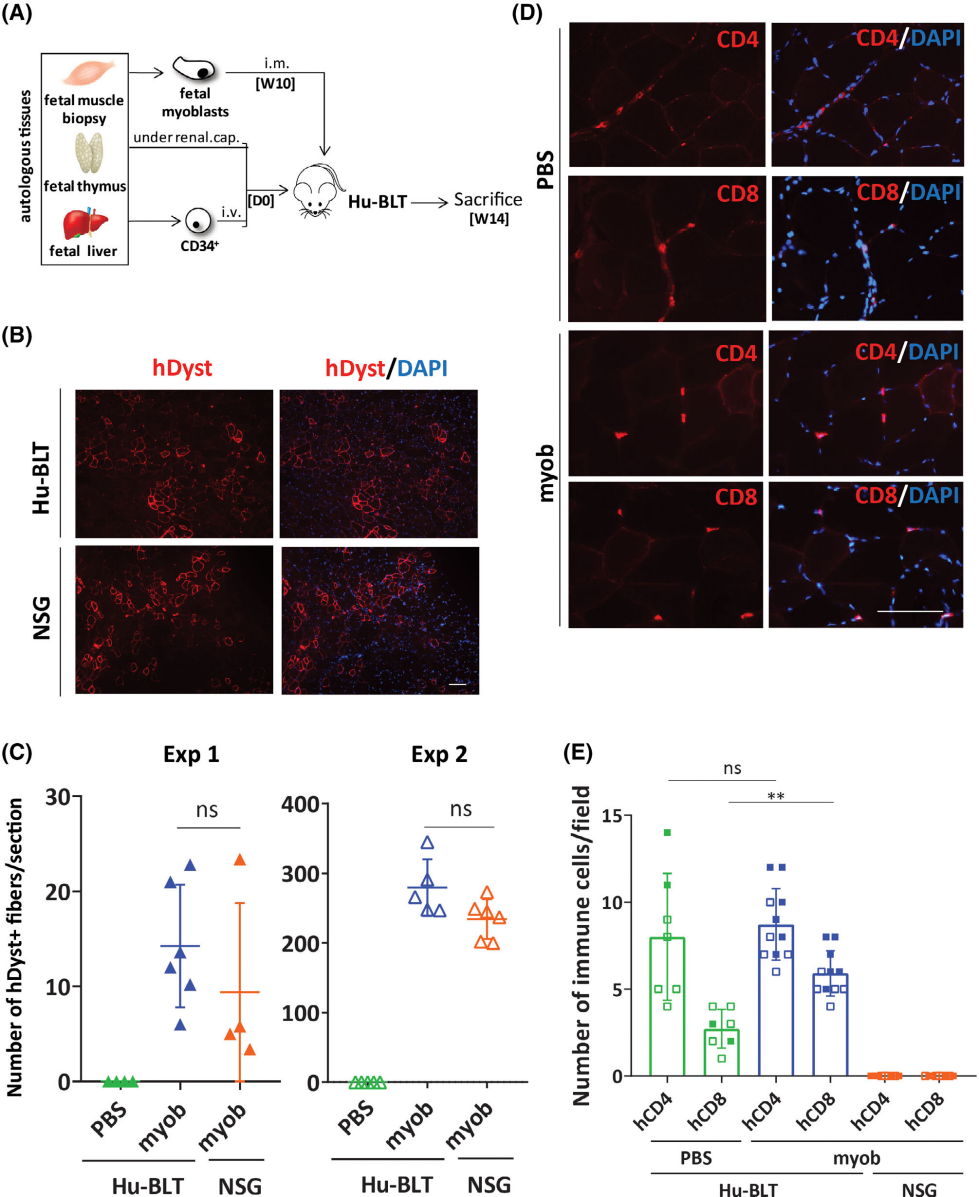
Infiltration of autologous T cells in muscles does not prevent engraftment of fetal myoblasts in Hu‐BLT mice. A, Schematic of the BLT humanized mouse model (Hu‐BLT). NSG mice were transplanted with human fetal thymus and autologous liver‐derived CD34^+^ cells. At week 10 (W10) postimmune reconstitution, human myoblasts isolated directly from a fetal muscle biopsy were injected in the skeletal muscle of Hu‐BLT. Mice were sacrificed 4 weeks later, and muscles were harvested and analyzed for cell engraftment and infiltration of immune cells. B, Representative photos of muscle sections from Hu‐BLT and NSG mice showing engraftment of autologous vs allogeneic human fetal myoblasts and resulting dystrophin positive fibers (in red). 4′,6‐Diamidino‐2‐phenylindole (DAPI) staining was performed to visualize nuclei (in blue). Showed are photos taken at ×10. Scale bar = 100 μm. C, Counts of human dystrophin‐positive fibers observed in muscle sections of Hu‐BLT mice transplanted with autologous fetal myoblast as shown in panel (B) or with phosphate buffered saline (PBS; sham). Also showed are counts in nonimmune reconstituted NSG mice to assess overall engraftment potential of the donor. Each dot represents the mean ± SEM number of dystrophin positive fibers from three selected sections in each muscle from an individual mouse. Total number of mice in both experiments is n = 11 Hu‐BLT, n = 10 NSG, and n = 7 Hu‐BLT sham (PBS only). D, Representative photos showing CD4 and CD8 T‐cell infiltration (in red) in muscle sections of Hu‐BLT mice either transplanted with autologous fetal myoblasts or injected with PBS (sham). DAPI staining was performed to visualize nuclei (in blue). Showed are photos taken at ×40. Scale bar = 100 μm. E, Frequency of CD4 and CD8 T cells infiltrated in muscles of Hu‐BLT and NSG mice. Each dot represents the number of T cells in four randomly selected fields in each muscle. n = 11 Hu‐BLT, n = 10 NSG, and n = 7 Hu‐BLT sham (PBS only). Note that full squares represent counts from experiment 1 and open squares counts from experiment 2

To evaluate the immunogenicity of skeletal muscle cell grafts, we used cells either obtained directly from a fetal muscle biopsy (myoblasts) or MPCs differentiated from fetal liver fibroblasts (Figures 1A and 2A). MPCs were generated using an efficient two‐step protocol leading to a high differentiation efficiency (100% of the cells expressing CD56) as we previously described.^11^ In brief, cells were first differentiated towards a mesenchymal‐like phenotype using myogenic media (see Methods) followed by the ectopic expression of MyoD using a nonreplicative type 5 adenoviruses containing the murine MyoD under a recombinant cytomegalovirus early enhancer/chicken β‐actin (CAG) promoter.^12^ We first confirmed that myoblasts and iPSC‐MPCs were positive for the myogenic markers CD56, CD82, Desmin and were fully capable of forming myotubes in vitro when placed under differentiation conditions (Figure S1). Immunogenicity was determined after cells were transplanted into the *Tibialis anterior* of Hu‐BLT mice showing high levels of reconstitution with either autologous or allogeneic immune cells (Figures 1A‐C and S2). Our results showed that 4 weeks after transplantation, muscle sections had a higher number of human dystrophin‐expressing myofibers resulting from the fusion of autologous compared to allogeneic MPCs (Figure 1B,C). As expected, muscles transplanted with allogeneic MPCs were highly infiltrated with CD4 and CD8 T cells (Figure 1D,E). Surprisingly, transplanted autologous muscles were also infiltrated with CD4 and CD8 T cells although at a much lower level (Figure 1E). Importantly, the engraftment of MPCs was not impaired by the infiltration of autologous T cells as an equal number of fibers expressing the human dystrophin were formed when MPCs were transplanted in nonreconstituted immune‐deficient NOD/SCID/IL2Rg null (NSG) mice (Figure 1C). Next, to demonstrate that lower engraftment in an allogenic condition was indeed mediated by immune cells, we transplanted allogeneic myoblasts into muscles of another group of Hu‐BLT mice treated or not with the immunosuppressive drug FK506. Mice were treated daily starting from the day of transplantation until the day of sacrifice for a total of 4 weeks. Our results showed that FK506 treatments prevented the rejection of allogeneic myoblasts which is consistent with a T cell‐dependent immune rejection mechanism (Figures S3A‐C). Moreover, an in vitro mixed lymphocyte reaction (MLR)‐like test showed that allogeneic but not autologous MPCs and myotubes were able to activate T cells after a tree day‐coculture with splenocytes collected from fully reconstituted Hu‐BLT mice (Figure 1F).

To determine if the low level infiltration of autologous T cells was specific to the differentiation procedure of MPCs, primary myoblasts isolated directly from a fetal muscle biopsy were transplanted into the *Tibialis anterior* of Hu‐BLT mice generated with thymus and hematopoietic cells obtained from the same donor as the myoblasts (Figure 2A). To our surprise, 4 weeks after cell transplantation, we also found muscles transplanted with autologous primary myoblasts to be infiltrated with CD4 and CD8 T cells at a level similar to what we observed using autologous MPCs (Figures 1D,E and 2D,E). Of note, sham injected muscles also had infiltrating CD4 and CD8 T cells suggesting low level inherent immune infiltration in muscle of Hu‐BLT mice. As observed with MPCs, the infiltration of T cells did not compromise engraftment of transplanted myoblasts (Figure 2B,C). Of note, we observed a striking increase in the number of engrafted human muscle fibers in experiment 2 compared to experiment 1, a phenotype unlikely to be explained by the inherent donor to donor variation. Instead, we believe such an increase is likely explained by the fact that MPCs from donor 2 were sorted for the expression of CD56 and that only the cells expressing the highest level were injected in mice (Figure S1B). Nonetheless, such an increase in engraftment levels did not translate into an increase number of immune cells infiltration (Figure 2E). These results suggest that infiltration of autologous T cells alone should not be considered as a predictive marker of successful engraftment in skeletal muscles.

### 3.2 hiPSC‐MPCs are not the target of autologous T and NK cells in vitro

**FIGURE 3:**
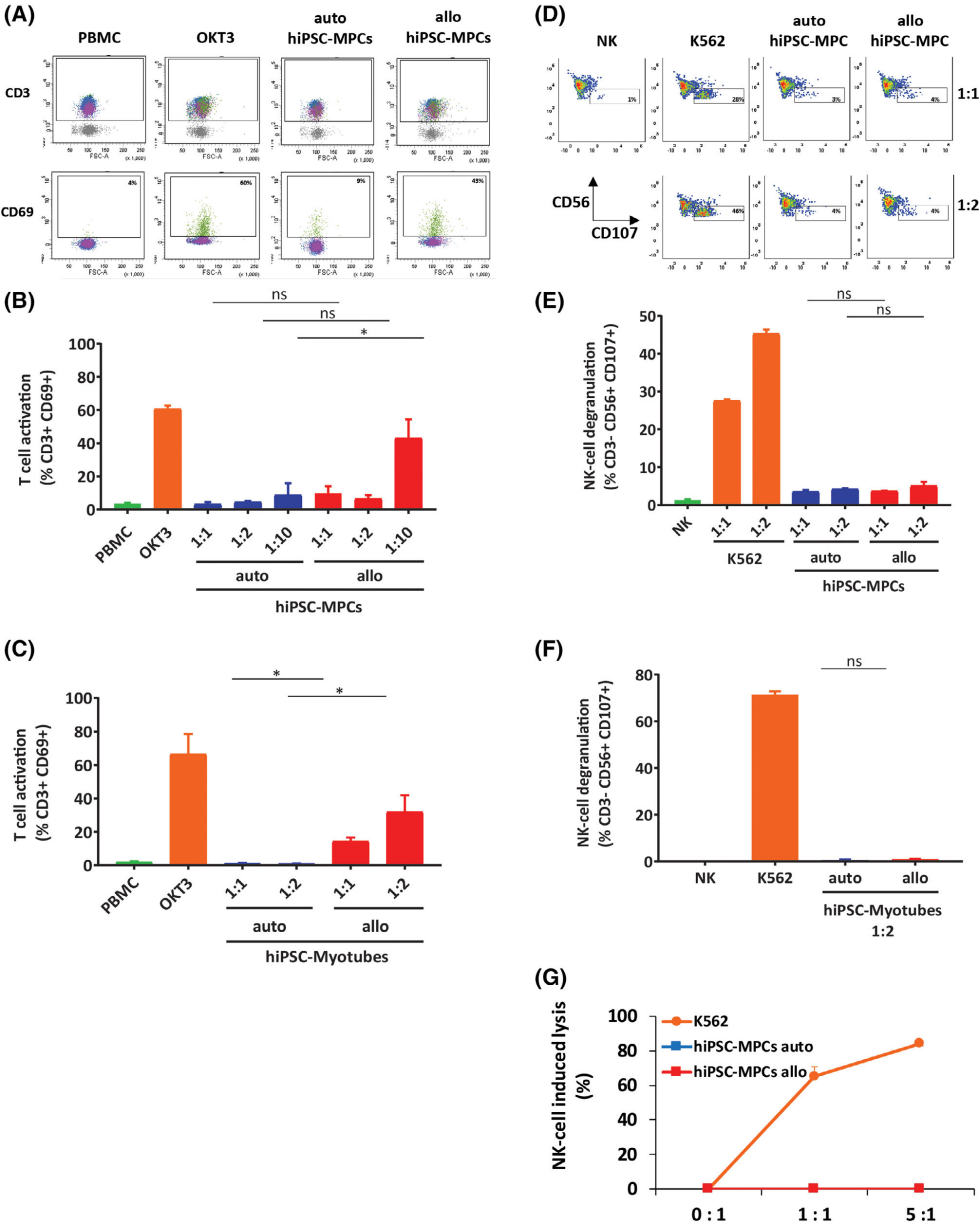
Myogenic progenitor cells derived from human iPSC are not the target of autologous immune cells in vitro. A‐C, hiPSC‐MPCs do not induce T‐cell activation in vitro. Representative flow cytometry plots (A) and measures of T‐cell activation (as determined by gating for CD3+/CD69+ cells) after coculture of hiPSC‐derived MPCs (B) or myotubes (C) with autologous or allogeneic peripheral blood mononuclear cells (PBMCs) for 3 days. OKT3 was used as a positive control. Shown is the mean ± SEM of two independent experiments done in triplicate using cells collected from two different donors. D‐F, hiPSC‐MPCs do not induce degranulation of NK cells in vitro. Representative flow cytometry plots (D) and measure of NK cell degranulation in vitro (as determined by evaluating CD107 expression on gated CD3^−^/CD56^+^ NK cell populations) after a 4 hour coculture between freshly isolated PBMCs and autologous or allogeneic hiPSC‐MPCs (E) or myotubes (F) at indicated ratios. K562 cells were used as a positive control. Shown is the mean ± SEM of two independent experiments done in triplicate using cells collected from two different donors. D, hiPSC‐MPCs were not lysed by purified NK cells in vitro. Cell lysis was determined by flow cytometry with the absolute count of PKH26‐stained hiPSC‐MPCs after a 4‐hour coculture with NK cells purified from autologous or allogeneic PBMCs by magnetic negative selection. K562 cells were used as a positive control. Shown is the mean ± SEM of two independent experiments done in triplicate using cells collected from two different donors. hiPSC, human induced pluripotent stem cell; iPSC, induced pluripotent stem cell; MPCs, myogenic progenitor cells; NK, natural killer

To better reflect future clinical settings, we then choose to differentiate MPCs from hiPSCs, instead of from fibroblasts and to evaluate their immunogenicity to T and NK cells in both autologous and allogeneic in vitro conditions. We first generated hiPSC clones using the integration‐free (Sendai virus) approach and confirmed they had normal karyotypes, expressed the classic markers of pluripotency and were able of forming in vivo teratoma in immune‐deficient mice (Figure S4A,B and data not shown), then we generated MPCs using the same two‐step differentiation protocol described above (Figure S1A). Using hiPSC‐MPCs, we then performed an MLR‐like assay using PBMCs and a cytotoxicity assay using PBMC‐purified NK cells. Our results show that hiPSC‐MPCs and their derived myotubes significantly activated allogeneic but not autologous T cells—even at the highest ratio (1:10) of PBMCs vs hiPSC‐MPCs (Figure 3A‐C). In addition, none of hiPSC‐MPCs or their derived myotubes were able to induce NK cell‐degranulation and/or specific cytotoxicity (Figure 3D‐G). This could be explained by the fact that hiPSC‐MPCs, in opposition to hiPSC, express high levels of HLA‐I as we have shown before.^13^

### 3.3 hiPSC‐derived myofibers are not rejected in Hu‐AT mice

**FIGURE 4:**
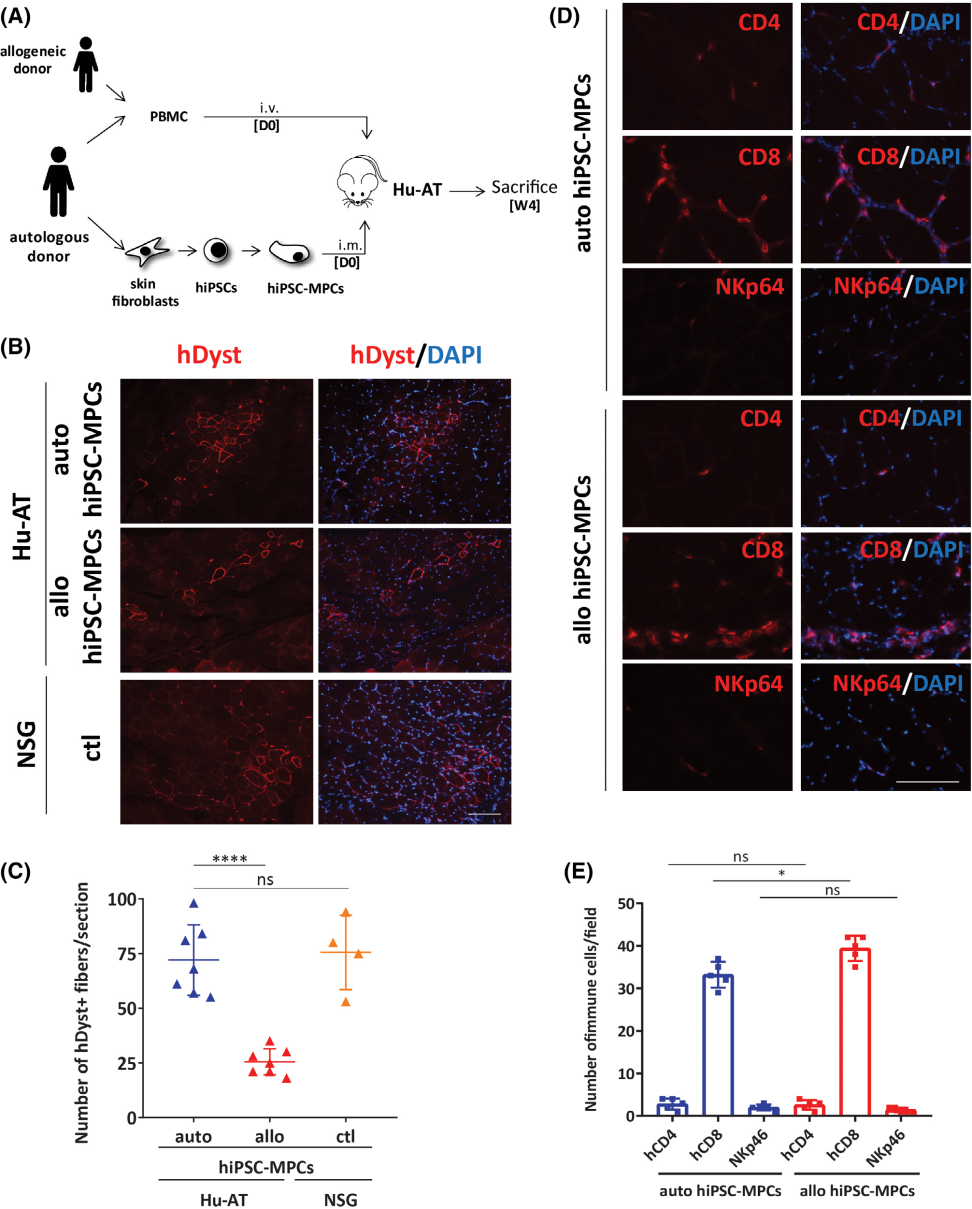
iPSC‐derived myogenic progenitor cells are immune tolerated in Hu‐AT mice. A, Schematic of the humanized mouse model generated following the AT (Hu‐AT) of human peripheral blood mononuclear cells (PBMCs). NSG‐SGM3 mice were transplanted on day 0 (D0) with hiPSC‐derived MPCs and either autologous or allogeneic PBMCs. Mice were sacrificed 4 weeks later, and skeletal muscles were harvested and muscle sections were analyzed for cell engraftment and infiltration of immune cells. B, Representative photos of muscle sections from Hu‐AT and NSG mice showing increased engraftment of autologous compared to allogeneic hiPSC‐MPCs and resulting dystrophin positive fibers (in red). 4′,6‐Diamidino‐2‐phenylindole (DAPI) staining was performed to visualize nuclei (in blue). Showed are photos taken at ×20. Scale bar = 100 μm. C, Counts of human dystrophin positive fibers observed in muscle sections of Hu‐AT mice transplanted with hiPSC‐MPCs as shown in panel (B). Also showed are counts in nonimmune reconstituted NSG mice to assess overall engraftment potential of transplanted cells. Each dot represents the mean ± SEM number of dystrophin positive fibers from five selected sections in each mouse. n = 7. D, Representative photos showing CD4, CD8 T cell and NKp46 NK cell infiltration (in red) in muscle sections of Hu‐AT mice transplanted with hiPSC‐MPCs and autologous or allogeneic PBMCs. DAPI staining was performed to visualize nuclei (in blue). Showed are photos taken at ×40. Scale bar = 100 μm. E, Frequency of CD4+, CD8+ T cells and NKp46^+^ NK cells infiltrated in muscles of Hu‐AT mice as shown in panel (D). Each dot represents the number of T cells in four randomly selected fields in each muscle. n = 5. hiPSC, human induced pluripotent stem cell; iPSC, induced pluripotent stem cell; MPCs, myogenic progenitor cells; NK, natural killer

To confirm our results showing the absence of NK‐mediated cytotoxicity in vitro and because Hu‐BLT mice are deficient in functional NK cells, we choose to further evaluate the immunogenicity of MPCs using a different humanized mouse model that combines functional T and NK cells. To this end, we adoptively transferred 1 × 10^7^ human PBMCs obtained from the same donors as the fibroblasts that were used to generate hiPSCs in NSG‐SGM3 mice (Figure 4A). We here choose to use NSG‐SGM3 mice based on previous work from our laboratory showing that AT of human PBMCs in these mice was highly effective at rejecting both autologous and allogenic tumor cells.^14^ Moreover, NSG‐SGM3 mice were shown to better support the engraftment of myeloid lineages that could play an important role in the first steps of the immune response.^15^ Hence, similar to what we observed in Hu‐BLT mice, 4 weeks post transplantation more dystrophin‐positive myofibers were observed in muscles of mice injected with autologous compared to allogeneic PBMCs (Figure 4B,C). Muscle tissues of both allogeneic and autologous mice were however highly infiltrated with CD8 T cells (Figure 4D,E). Yet, the infiltration of autologous CD8 T cells had surprisingly no impact on the graft as the number of hiPSC‐MPCs‐derived myofibers was not higher in non‐reconstituted NSG‐SGM3 mice (Figure 4C). Infiltration of CD8 T cells was likely not specific to grafted human myofibers as noninjected muscle sections were also heavily infiltrated with CD8 T cells suggesting mice were at an early stage of a full xenograft reaction (data not shown). Overall, our results demonstrate that hiPSC‐derived muscular grafts are likely to be tolerated by autologous T cells, rejected by allogeneic T cells and not the target of NK cells.

## 4 DISCUSSION

The success of future hiPSC‐derived cell therapies, whether obtained from autologous donors or through the development of universal cell lines, will depend on the ability to evade immune recognition.^16^, ^17^, ^18^ Using two distinct humanized mouse models, we here provide evidence that MPCs are tolerated by autologous T cells in both Hu‐BLT and Hu‐AT models and by NK cells in Hu‐AT mice. The fact that MPCs were not rejected by NK cells in Hu‐AT mice cannot be explain by an insufficient number of NK cells injected. Indeed, we recently showed the injection of a similar number of NK cells was sufficient to prevent the growth of iPSC‐derived teratomas in the same model.^13^

Moreover, our observation in Hu‐BLT mice that MPCs attract autologous CD4 and CD8 T cells at a level similar to that of myoblasts derived from a muscle biopsy, yet without compromising engraftment, was surprising. Our hypothesis is that T cells may have been attracted by an altered secretory phenotype acquired during the short in vitro growth expansion period of MPCs and myoblasts. Another possibility is that aberrantly expressed antigens may have attracted and induced tolerance/exhaustion of infiltrating autologous T cells. Unfortunately, the low level of infiltrating T cells did not allow to test this hypothesis. It is also important to keep in mind that the capacity of iPSC‐derived MPCs to arouse an immune response is dependent on the differentiation protocol used and that other differentiation protocols available for the generation of MPCs from iPSCs will also need to be evaluated for their ability to evade the immune system.^19^, ^20^, ^21^ It also remains to be determined if the presence of autologous T cells can affect long‐term engraftment (more than 4 weeks). Indeed, 4 weeks after the AT of PBMCs, we observed equal infiltration of T cells in both autologous and allogeneic conditions in the Hu‐AT model (Figure 4E), suggesting mice were at the early stage of graft‐vs‐host disease. The use of mice that lack the murine MHC allowing to evade graft‐vs‐host disease but that retain T cell function upon engraftment will likely help to overcome this issue.^22^ Our results also provide the first evidence that hiPSC‐derived MPCs, unlike hiPSCs, are not the target of NK cells in vivo. Overall, our study provides essential preclinical data supporting the usage of fibroblasts or iPSC‐derived MPCs in regenerative medicine.

## CONFLICT OF INTEREST

The authors declared no potential conflicts of interest.

## AUTHOR CONTRIBUTIONS

B.B: conception and design, experiment performing, collection and assembly of data, data analysis and interpretation, manuscript writing; C.D.‐L., M.‐L.G., C.C., Y.L., C.M.: experiment performing; J.V.G.: provision of study material; J.P.T.: provision of study material and expertise; E.H.: conception and design; C.B.: conception and design, data analysis and interpretation, manuscript writing.

## Supporting information


**Figure S1** **Myogenic progenitor cell differentiation and characterization. (A)** Schematic illustration of the protocol used for the differentiation of fibroblasts and hiPSCs in MPCs using myogenic medium (MB1) and a MyoD‐expressing adenoviral vectors. Flow cytometry plots showing the increased expression of the myogenic markers CD56 and CD82 in differentiated cells after MyoD expression (in blue). IgG isotype controls are also shown (in red). **(B)** Flow cytometry analysis of CD56 and CD82 expression on fetal myoblasts before and after CD56‐based cell sorting. IgG isotype controls are also shown (in red). **(C)** Representative photos showing expression of the myogenic cell markers Desmin, Myogenin or the Myosin Heavy Chain (in red) on iPSC‐MPCs and biopsy‐derived fetal myoblasts compared to skin fibroblasts. DAPI staining was performed to visualize nuclei (in blue). **(D)** Phenotypic characterization of hiPSC‐ derived MPCs and biopsy‐derived fetal myoblasts. Cells were stained with the indicated mAbs (in black) or IgG isotype controls (in white) and analyzed by flow cytometry. Acquisition from one representative experiment is shown for MPCs (top panel) and fetal myoblasts (lower panel).
**Figure S2. Immune reconstitution in Hu‐BLT mice. (A)** Immune reconstitution of a Hu‐BLT mouse 13 weeks following the transplantation of CD34+ fetal liver cells and autologous thymic tissues. Representative plots of human T cells (CD3, CD4) and B cells (CD19) reconstitution in peripheral blood are shown. **(B)** Proportion of the major leucocytes subsets found in the peripheral blood of representative Hu‐BLT mice 13 weeks following their reconstitution. First, hCD45 expressing cells were gated to estimate the total level of engrafted human cells then the percentage of T cells (hCD3), and B cells (hCD19) were determined among hCD45+ cells. The proportion of hCD4+ cells is shown as the percentage among hCD3+ cells. **(C)** Representative photos of a human thymic (T) implant under the mouse renal capsule (K). Also showed are representative thymus sections showing human T cells (CD3, CD4 and CD8 in red). DAPI staining was performed to visualize nuclei (in blue).
**Figure S3. Tacrolimus‐based immune‐rejection blockade in Hu‐BLT mice. (A)** Schematic of the myoblast transplantation in the skeletal muscle of Hu‐BLT mice and their immune suppression using FK506. In brief, Hu‐BLT mice were generated as previously described and were transplanted with allogeneic myoblasts isolated from a biopsy at week 15 post immune reconstitution. Myoblasts were modified to express the green fluorescent protein (GFP) before transplantation. Mice received daily injections of FK506 or PBS (as a sham) starting immediately following the transplantation of myoblasts until sacrifice at week 18 (W18). **(B)** Representative photos of the whole muscle section from Hu‐BLT mice treated either with PBS or with FK506 showing increased engraftment of allogeneic myoblasts under immunosuppression as determined by the high number of GFP positive myofibers resulting from the fusion of transplanted myoblasts (in green).). Showed are photos taken at 20X. Scale bar, 100 μm. **(C)** Representative photos showing decreased CD8 T cell infiltration (in red) in muscle sections of Hu‐BLT treated with FK506. DAPI staining was performed to visualize nuclei (in blue). Showed are photos taken at 20X. Scale bar, 100 μm.
**Figure S4. hiPSC characterization. (A)** Representative photos showing the expression of pluripotency markers (Tra1‐60, OCT4 in red and Sox2, SSEA4 in green) in skin fibroblast‐derived hiPSCs. Cells were cultured in feeder‐free conditions on Geltrex‐coated dishes in E8 medium and passaged every 3‐4 days. Shown in blue are nuclei stained with DAPI. **(B)** Hematoxylin and eosin staining of a teratoma‐derived from 1x106 cells of one hiPSC clone at passage 17 injected under the renal capsule of a NSG mouse and grown during 8 weeks. Representative photos showing tissues from the three embryonic germ layers are shown. 10X.
Table 1: List of antibodies.

## Data Availability

The data that support the findings of this study are available on request from the corresponding author.
