# Supplementary material for: Myogenic progenitor cells derived from human induced pluripotent stem cell are immune‐tolerated in humanized mice
Source: Stem Cells Transl Med. 2020 Sep 2;10(2):267–77. doi: 10.1002/sctm.19-0452 (PMC7848353; doi:10.1002/sctm.19-0452)
Supplement: Supplementary file 1 — Figure S1 Myogenic progenitor cell differentiation and characterization. (A) Schematic illustration of the protocol used for the differentiation of fibroblasts and hiPSCs in MPCs using myogenic medium (MB1) and a MyoD‐expressing adenoviral vectors. Flow cytometry plots showing the increased expression of the myogenic markers CD56 and CD82 in differentiated cells after MyoD expression (in blue). IgG isotype controls are also shown (in red). (B) Flow cytometry analysis of CD56 and CD82 expression on fetal myoblasts before and after CD56‐based cell sorting. IgG isotype controls are also shown (in red). (C) Representative photos showing expression of the myogenic cell markers Desmin, Myogenin or the Myosin Heavy Chain (in red) on iPSC‐MPCs and biopsy‐derived fetal myoblasts compared to skin fibroblasts. DAPI staining was performed to visualize nuclei (in blue). (D) Phenotypic characterization of hiPSC‐ derived MPCs and biopsy‐derived fetal myoblasts. Cells were stained with the indicated mAbs (in black) or IgG isotype controls (in white) and analyzed by flow cytometry. Acquisition from one representative experiment is shown for MPCs (top panel) and fetal myoblasts (lower panel). Figure S2. Immune reconstitution in Hu‐BLT mice. (A) Immune reconstitution of a Hu‐BLT mouse 13 weeks following the transplantation of CD34+ fetal liver cells and autologous thymic tissues. Representative plots of human T cells (CD3, CD4) and B cells (CD19) reconstitution in peripheral blood are shown. (B) Proportion of the major leucocytes subsets found in the peripheral blood of representative Hu‐BLT mice 13 weeks following their reconstitution. First, hCD45 expressing cells were gated to estimate the total level of engrafted human cells then the percentage of T cells (hCD3), and B cells (hCD19) were determined among hCD45+ cells. The proportion of hCD4+ cells is shown as the percentage among hCD3+ cells. (C) Representative photos of a human thymic (T) implant under the mouse renal capsule (K) [file SCT3-10-267-s001.pdf]

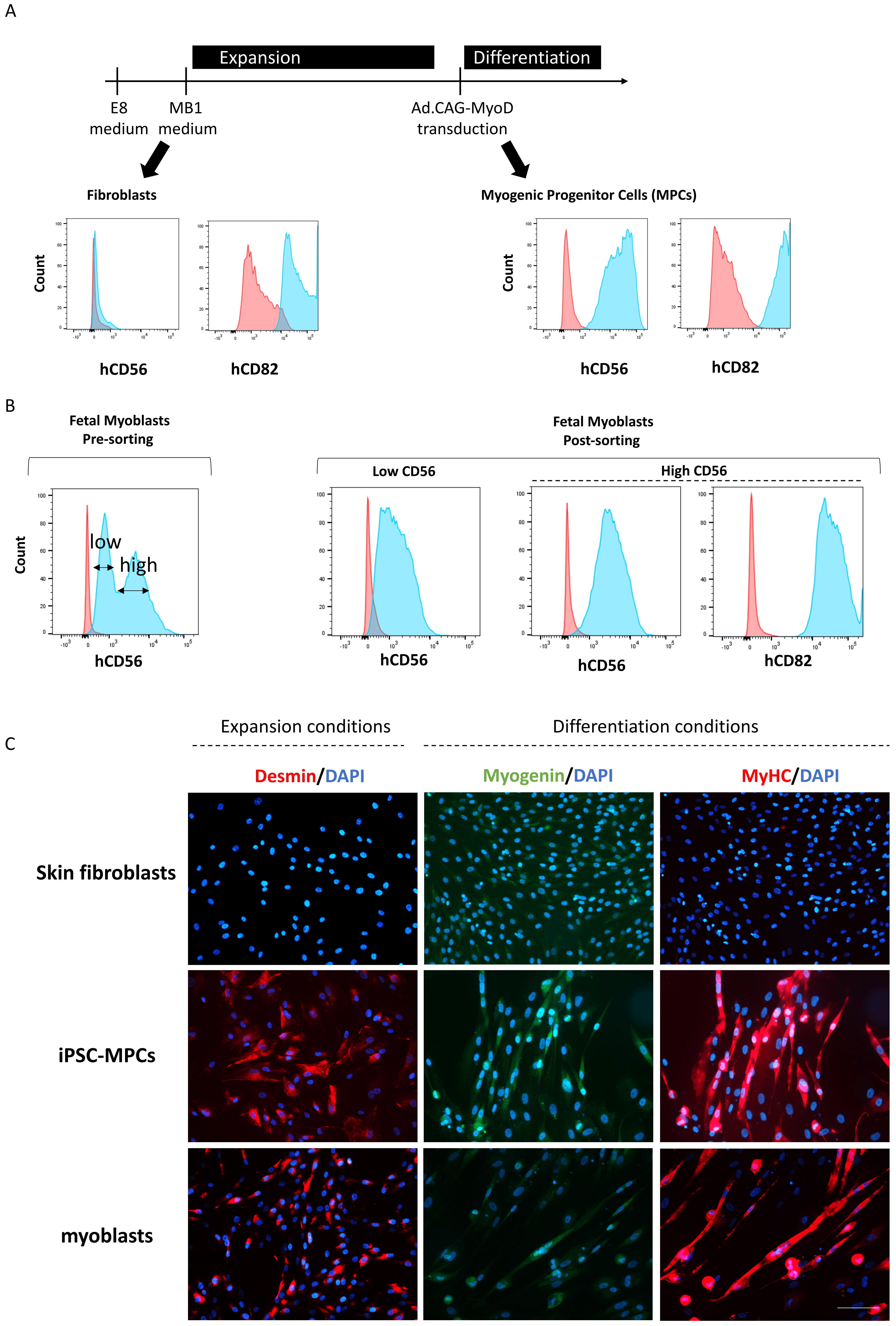

Benabdallah et al. Figure S1

D

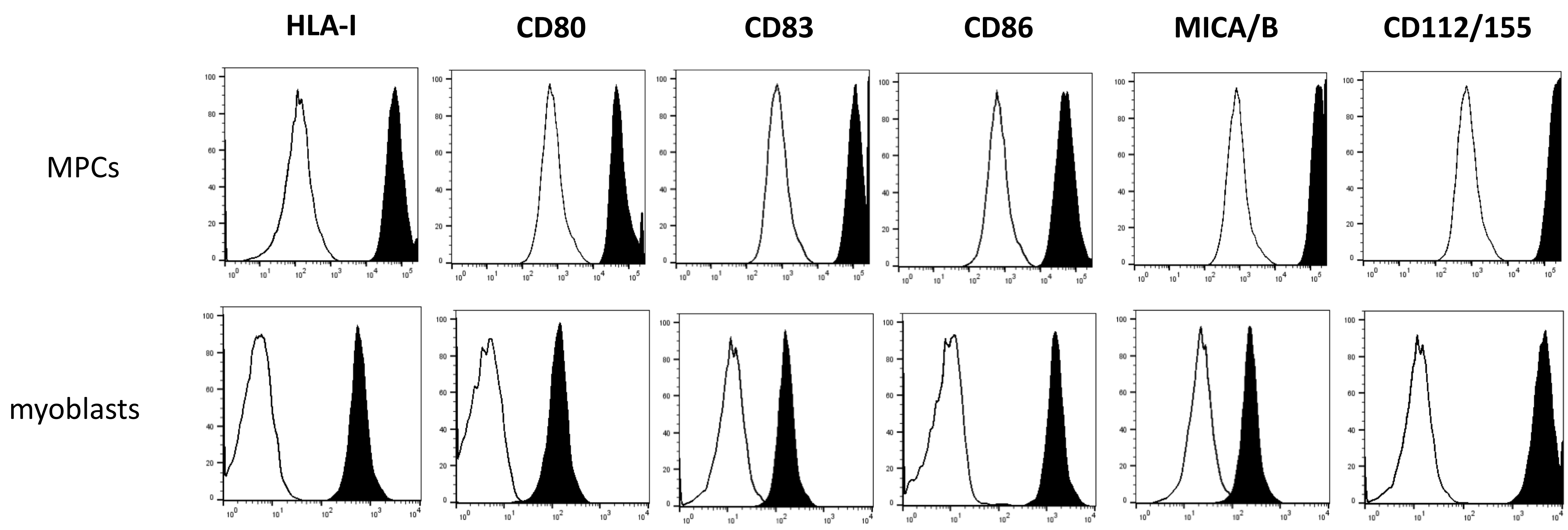

Figure S1. Myogenic progenitor cell differentiation and characterization.

(A) Schematic illustration of the protocol used for the differentiation of fibroblasts and hiPSCs in MPCs using myogenic medium (MB1) and a MyoD-expressing adenoviral vectors. Flow cytometry plots showing the increased expression of the myogenic markers CD56 and CD82 in differentiated cells after MyoD expression (in blue). IgG isotype controls are also shown (in red).

(B) Flow cytometry analysis of CD56 and CD82 expression on fetal myoblasts before and after CD56-based cell sorting. IgG isotype controls are also shown (in red).

(C) Representative photos showing expression of the myogenic cell markers Desmin, Myogenin or the Myosin Heavy Chain (in red) on iPSC-MPCs and biopsy-derived fetal myoblasts compared to skin fibroblasts. DAPI staining was performed to visualize nuclei (in blue).

(D) Phenotypic characterization of hiPSC- derived MPCs and biopsy-derived fetal myoblasts. Cells were stained with the indicated mAbs (in black) or IgG isotype controls (in white) and analyzed by flow cytometry. Acquisition from one representative experiment is shown for MPCs (top panel) and fetal myoblasts (lower panel).

A

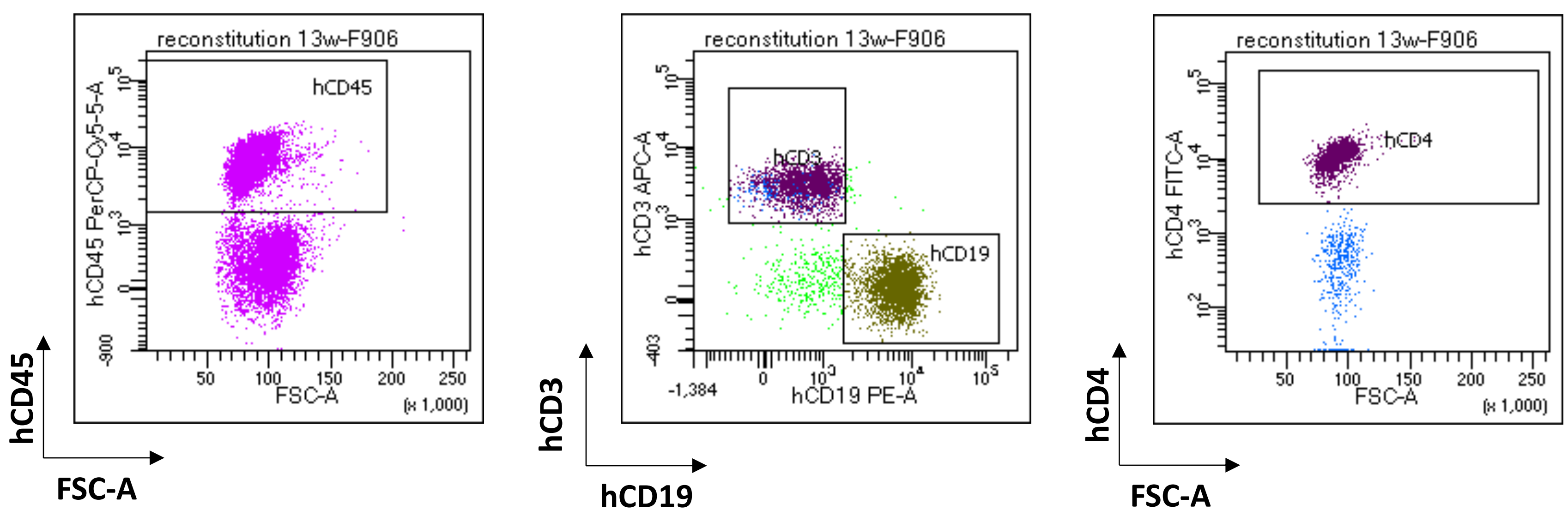

B

Hu-BLT Mice	% hCD45	% hCD19	% hCD3	% hCD4
1	51	16	81	72
2	65	19	76	72
3	39	25	71	70
4	39	37	57	74
5	61	26	68	69

C

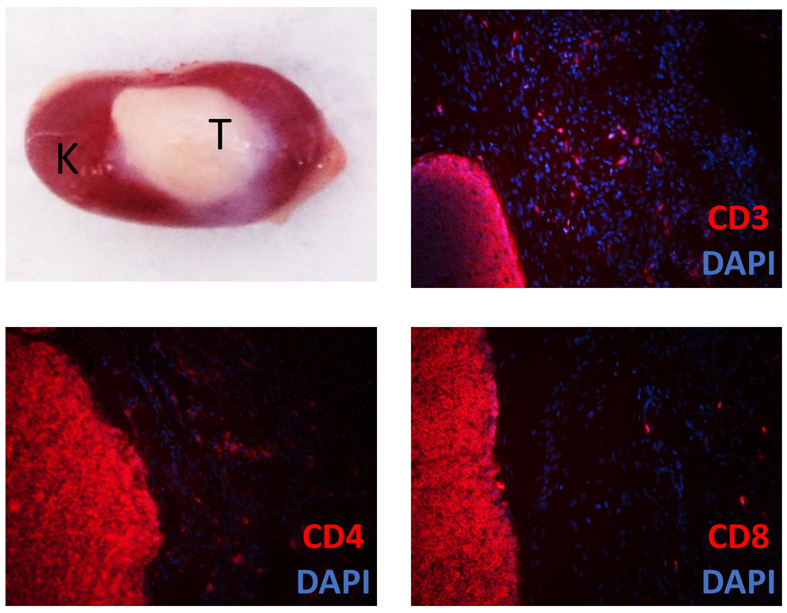

Figure S2. Immune reconstitution in Hu-BLT mice.

(A) Immune reconstitution of a Hu-BLT mouse 13 weeks following the transplantation of $CD34^{+}$ fetal liver cells and autologous thymic tissues. Representative plots of human T cells (CD3, CD4) and B cells (CD19) reconstitution in peripheral blood are shown.

(B) Proportion of the major leucocytes subsets found in the peripheral blood of representative Hu-BLT mice 13 weeks following their reconstitution. First, hCD45 expressing cells were gated to estimate the total level of engrafted human cells then the percentage of T cells (hCD3), and B cells (hCD19) were determined among hCD45+ cells. The proportion of hCD4+ cells is shown as the percentage among hCD3+ cells.

(C) Representative photos of a human thymic (T) implant under the mouse renal capsule (K). Also showed are representative thymus sections showing human T cells (CD3, CD4 and CD8 in red). DAPI staining was performed to visualize nuclei (in blue).

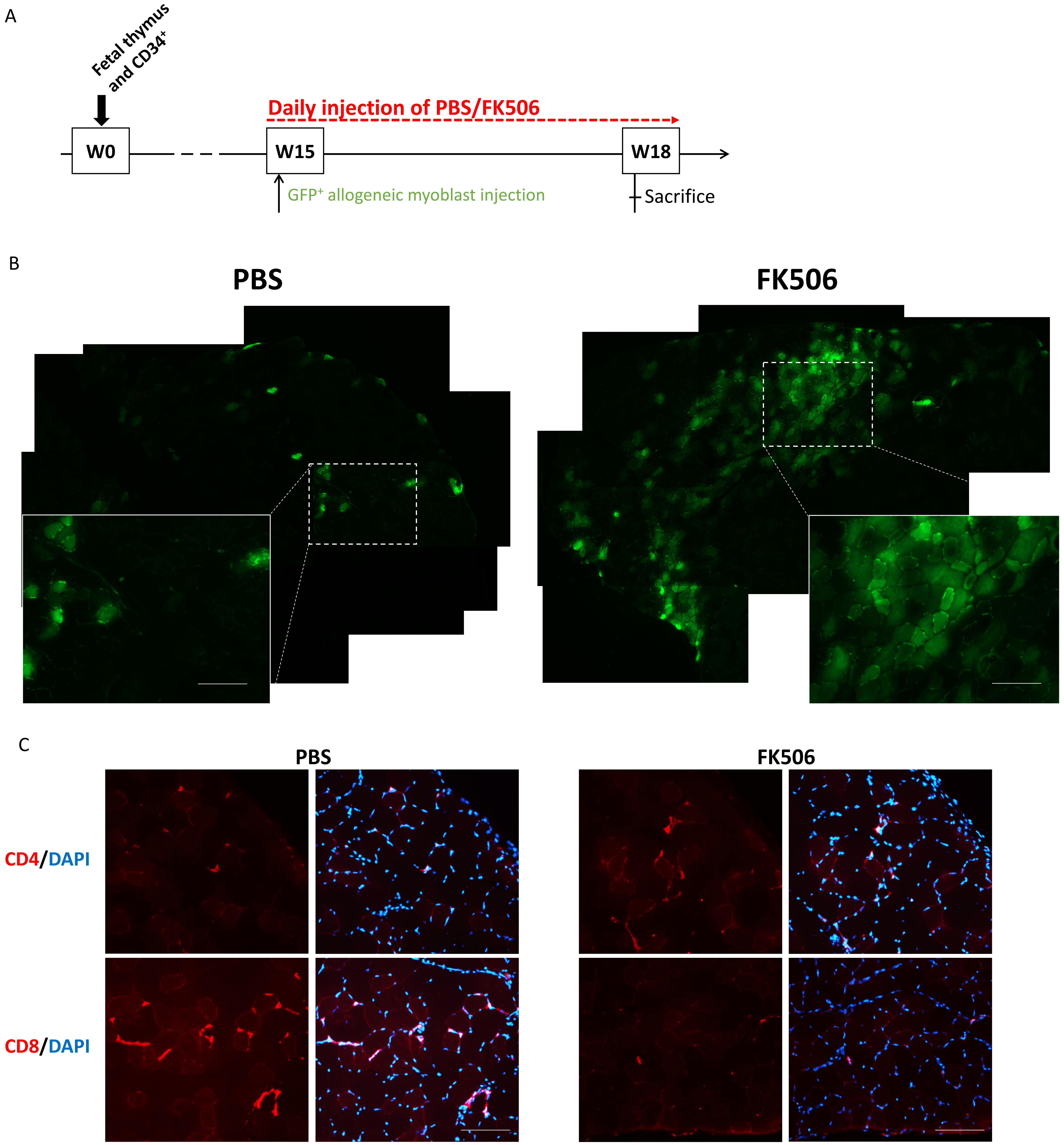

Figure S3. Tacrolimus-based immune-rejection blockade in Hu-BLT mice.

(A) Schematic of the myoblast transplantation in the skeletal muscle of Hu-BLT mice and their immune suppression using FK506. In brief, Hu-BLT mice were generated as previously described and were transplanted with allogeneic myoblasts isolated from a biopsy at week 15 post immune reconstitution. Myoblasts were modified to express the green fluorescent protein (GFP) before transplantation. Mice received daily injections of FK506 or PBS (as a sham) starting immediately following the transplantation of myoblasts until sacrifice at week 18 (W18).

(B) Representative photos of the whole muscle section from Hu-BLT mice treated either with PBS or with FK506 showing increased engraftment of allogeneic myoblasts under immunosuppression as determined by the high number of GFP positive myofibers resulting from the fusion of transplanted myoblasts (in green).). Showed are photos taken at 20X. Scale bar, 100 µm.

(C) Representative photos showing decreased CD8 T cell infiltration (in red) in muscle sections of Hu-BLT treated with FK506. DAPI staining was performed to visualize nuclei (in blue). Showed are photos taken at 20X. Scale bar, 100 µm.

Benabdallah et al. Figure S3

A

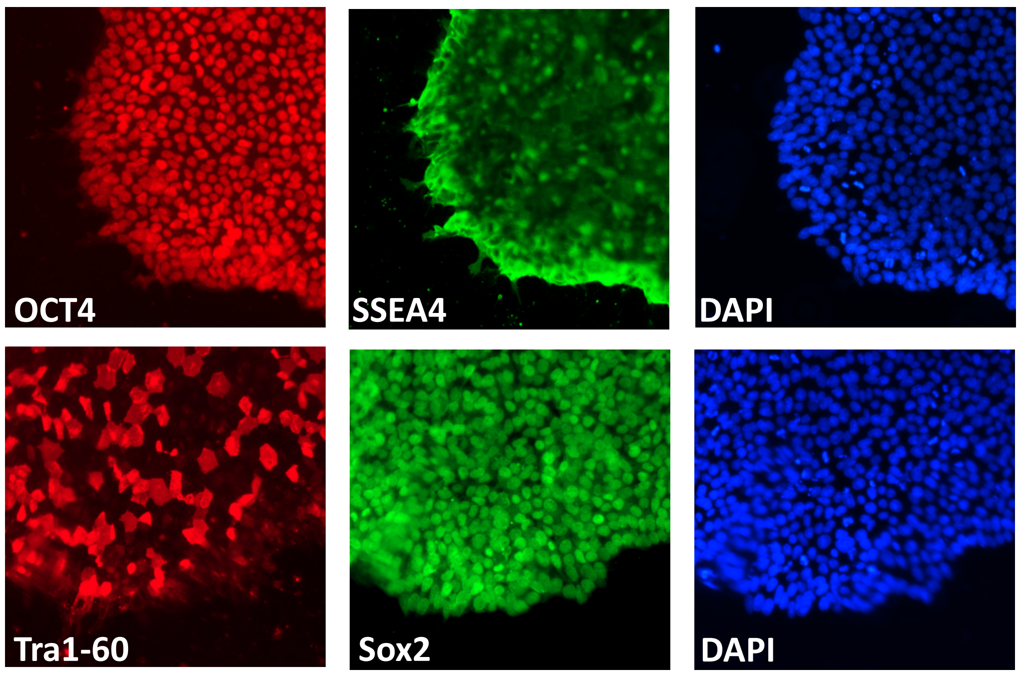

B

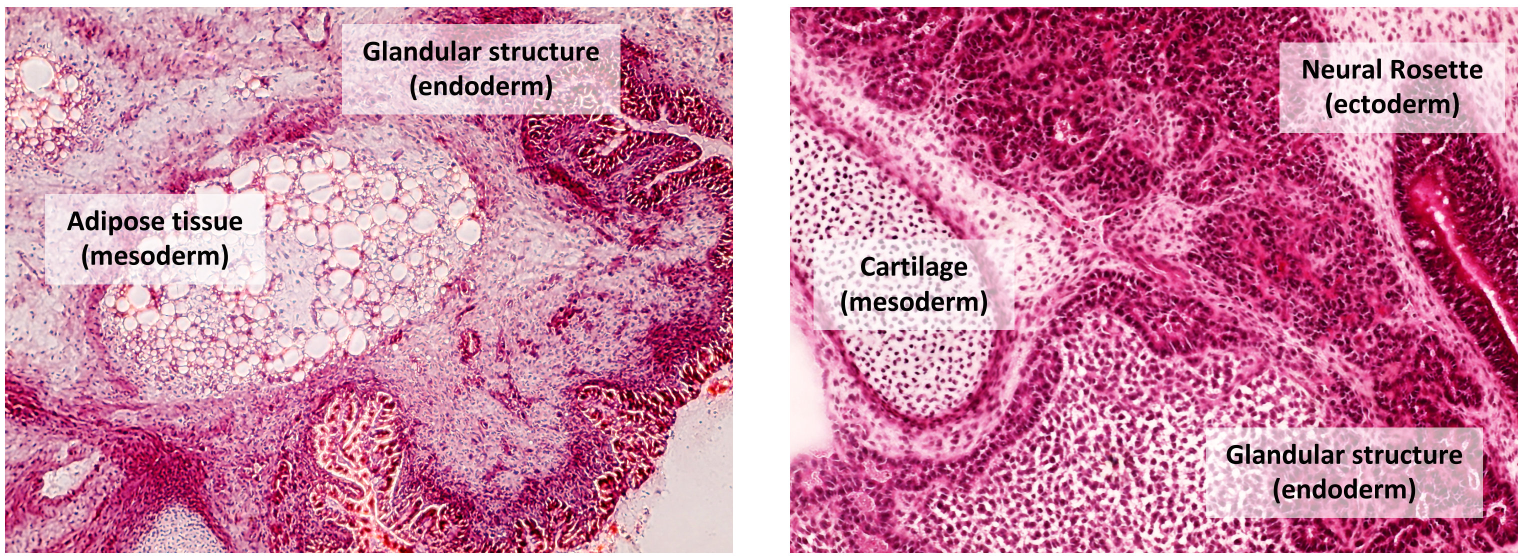

Figure S4. hiPSC characterization.
(A) Representative photos showing the expression of pluripotency markers (Tra1-60, OCT4 in red and Sox2, SSEA4 in green) in skin fibroblast-derived hiPSCs. Cells were cultured in feeder-free conditions on Geltrex-coated dishes in E8 medium and passaged every 3-4 days. Shown in blue are nuclei stained with DAPI.
(B) Hematoxylin and eosin staining of a teratoma-derived from $1x10^6$ cells of one hiPSC clone at passage 17 injected under the renal capsule of a NSG mouse and grown during 8 weeks. Representative photos showing tissues from the three embryonic germ layers are shown. 10X.

Table 1: List of antibodies.

Antibodies	Clones	Catalog #	Suppliers
APC-hCD56	HCD56	318309	Biolegend
PE-hCD82	ASL-24	342103	Biolegend
PerCP-Cy5.5-hCD45	HI30	304014	Biolegend
APC-hCD3	UCHT1	300412	Biolegend
PE-hCD19	HIB19	302208	Biolegend
FITC-hCD4	A161A1	357406	Biolegend
PE-hCD69	FN50	310906	Biolegend
Purified anti-human CD3	OKT3	317301	Biolegend
FITC-hCD107a	5123804	555800	BD Biosciences
FITC-hCD107b	4341653	555804	BD Biosciences
Purified anti-human CD4	RPA-T4	300502	Biolegend
Purified anti-human CD8	HIT8a	300901	Biolegend
Purified anti-human NKp46/NCR1	195314	AF1850	R&D systems
BV-711-hHLA-ABC	G46-2.6	565333	BD Biosciences
PE-Cy7-hCD80	2D10	205207	Biolegend
PE-hCD83	HB15e	305307	Biolegend
APC-hCD86	B70	305411	Biolegend
APC-hMICA/B	6D4	320907	Biolegend
PE-hCD155	SKIL4	337609	Biolegend
PE-hCD112	TX31	337409	Biolegend
Purified anti-human OCT4	74761074A	A24867	Life Technologies
Purified anti-human SSEA4	RB232365E	A24866	Life Technologies
Purified anti-human SOX2	AL769A	A24759	Life Technologies
Purified anti-human TRA-1-60	RF234864D	A24868	Life Technologies
Purified anti-Desmin	D33	M0760	DAKO
Purified anti MyHC	MF20	MF20	DHSB
Purified anti-Myogenin	EPR4789	ab124800	Abcam
